# Luminescent and Optically Detected Magnetic Resonance Studies of CdS/PVA Nanocomposite

**DOI:** 10.1186/s11671-017-1892-4

**Published:** 2017-02-20

**Authors:** Galyna Yu. Rudko, Igor P. Vorona, Volodymyr I. Fediv, Andrii Kovalchuk, Jan E. Stehr, Bela D. Shanina, WeiMin M. Chen, Irina A. Buyanova

**Affiliations:** 1 V. Lashkaryov Institute of Semiconductor Physics of National Academy of Sciences of Ukraine, 45, Pr. Nauky, Kiev, 03028 Ukraine; 2 Department of Biophysics and Medical Informatics, Bukovinian State Medical University, 42 Kobylyanska st., Chernivtsi, 58000 Ukraine; 3 Department of Physics, Chemistry and Biology, Linköping University, SE-581 83 Linköping, Sweden

**Keywords:** Composite, Polymer, CdS nanoparticles, Photoluminescence, Interfacemagnetic resonance

## Abstract

A series of solid nanocomposites containing CdS nanoparticles in polymeric matrix with varied conditions on the interface particle/polymer was fabricated and studied by photoluminescence (PL) and optically detected magnetic resonance (ODMR) methods. The results revealed interface-related features in both PL and ODMR spectra. The revealed paramagnetic centers are concluded to be involved in the processes of photo-excited carriers relaxation.

## Background

Rapid development of physics and technology of nanosized objects gave birth to numerous new materials. Among them, the solid polymer-based nanocomposites containing either nanoparticles (NPs) or nanowires are continuously attracting great attention of researchers. The interest is caused by unique mechanical, optical, electrical, and magnetic properties of nanocomposites (see, e.g., the reviews [1–3] and references therein). These materials are promising in potential technological applications in the fields of optoelectronics, photonics, biosensing, etc. (see [4–6] and references therein). The source of nanocomposites uniqueness is that these materials can combine the useful properties of their organic and inorganic components. Moreover, new properties which do not exist for either component can arise from chemical bonding of NPs with matrix and also from energy or charge transfer between organic and inorganic parts of a composite [7–10]. Because of the nanoscale regime of NPs and a huge total area of their surface (which could be hundreds of square meters per cm^3^), the state of the interface—the thin layer between organic matrix and inorganic NPs—can play a key role in the formation of new emerging characteristics.

The state of an as-grown NP surface is predetermined by the conditions of solution phase of utilized synthetic methods. The latter should provide the restriction of the NPs final size, stabilization against agglomeration, and abruption of chemical reactions in the ready colloid. All these demands are satisfied by covering the NPs with surfactant molecules, or by using steric stabilization in the case when NPs are grown in polymeric environments, or by combination of both. The state of the NP surface can further be altered by multiple chemical and physical treatments. The post-growth surface modification methods include [11] the replacement of original surface-attached molecules by a variety of other chemical moieties, functionalization of NPs with various ligand molecules, coverage of a NP core with either inorganic or organic shell, transfer of NPs from aqueous to organic solutions, formation of physical mixtures of as-grown NPs with various polymers, etc. All these strategies involve the changes of the chemical environment of NPs, which leads to the formation of new chemical species on the NPs surface. This makes the analysis of surface-related phenomena rather complicated.

In the present study, we applied a simple way to modify interfacial conditions that does not involve either new chemical surrounding of unchanged NPs or formation of a shell around a NP. The idea is to change the degree of the NP surface saturation with polymeric molecules by varying the ratio between the synthesized NPs and macromolecules in the sample. We studied solid nanocomposites formed from the colloids CdS/polymer that were grown by one-pot procedure. The choice of the composite was dictated, on the one hand, by the known bright emission of CdS nanoparticles in the visible range and, on the other hand, by the possibility to grow NPs directly in the solution of polyvinyl alcohol (PVA) that forms a solid matrix of nanocomposite after drying. We used photoluminescence (PL) to study light-emitting properties of composites and optically detected magnetic resonance (ODMR) method to find out paramagnetic centers that participate in light emission processes.

## Methods

### Procedure of Nanocomposite Synthesis

Nanocomposites CdS/PVA were obtained by colloidal chemical route, i.e., CdS nanoparticles were synthesized directly in the water solution of PVA. Polymer molecules served as a capping agent that restricted the NPs size during the growth. The concentration of the starting polymer solution was 5 (wt.)%. The precursors for NPs growth—CdCl_2_ and Na_2_S salts—were step-wise added to the growth solution. In order to avoid undesirable byproducts, especially, to prevent the formation of Cd(OH)_2_, the overall amount of precursors and pH value of the solution were calculated as described in [12]. Each synthesis step was done at ambient conditions. When all chemical reactions in the solution were completed, the stable colloidal solution was formed. An average size of CdS NPs was 5.4 ± 0.4 nm (the transmission electron microscope image of these particles can be found in [9]). The final colloidal solutions were very stable; they did not change their properties for at least 3 years during the storage in a drawer at room temperature.

The series of colloids for further preparation of solid nanocomposites was obtained by diluting the final colloid with pure PVA solution. The resulting proportion of NPs concentration in these solutions was 1:0.5:0.25. This process implied only simple physical mixing and no additional chemicals were used. Thus, we assume that the ripened NPs in the colloid cannot be affected by this procedure, and their sizes remain unaltered while the coverage of NPs with macromolecules can be changed due to an excess of the added polymer.

Equal amounts of the diluted colloids were dried at ambient conditions in the closed vessels with absorbent to produce solid films of nanocomposites consisting of solid PVA matrix with embedded CdS NPs. The reference sample was formed from the native PVA solution by the same drying procedure. The concentrations of NPs in nanocomposites were 4°10^16^, 2°10^16^, and 1°10^16^ cm^−3^, correspondingly, the composites with the highest, middle, and lowest NPs concentrations were labeled H, M, and L. The reference sample of the unloaded polymer was labeled PVA. The content of the semiconductor phase in the solid film of the H composite is 0.5 vol%. An average thickness of the samples is about 250 μm.

### Experimental Techniques

Photoluminescence (PL) of nanocomposites as well as of pure PVA was excited with a Coherent Verdi 2 W laser. The excitation wavelength was 266 nm. PL spectra were recorded by a Princeton Instruments ST-133 CCD camera that is attached to a 0.5-m Acton SpectraPro 2500i monochromator. Measurements were done at low temperatures in a helium cryostat.

ODMR measurements were performed with a modified Bruker ER 200 D X band (microwave frequency ~9.3 GHz) in a liquid helium flow cryostat. A laser with the excitation wavelength of 351 nm was used for the PL excitation. A spectral range being necessary for the ODMR registration was selected by suitable optical filters. An ODMR signal was detected using a lock-in technique as a change of the PL intensity due to on-off modulation of a microwave power as a function of a magnetic field. The typical microwave power employed was 200 mW. The measurements were performed at 5 K.

## Results

### PL Spectra of Nanocomposites

**Fig. 1:**
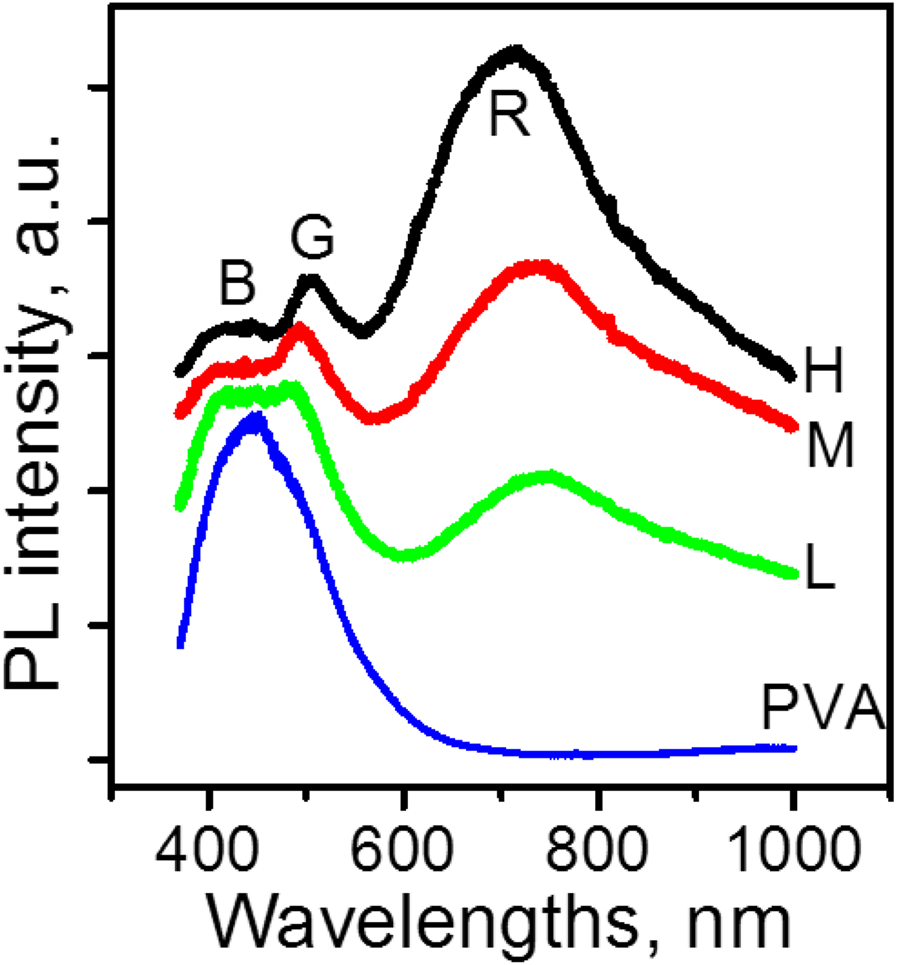
Photoluminescence spectra of the samples *L*, *M*, and *H* composites and pure PVA

Figure 1 shows PL spectra of the composites with different concentrations of NPs. The corresponding spectra are labeled H, M, and L. The spectrum of the pure PVA is also shown as a reference (labeled PVA). The latter consists of one asymmetric wide band with the maximum at 465 nm. The shape of the observed spectrum is typical for this polymer [13, 14] and was ascribed to carbonyl-related chromophore groups that are inherent to PVA.

At least three spectral features are distinguishable in the PL spectra of composites: two PL bands in the blue-green spectral range (~400–550 nm) and an extremely broad band covering the red and near infrared spectral ranges (~550–1000 nm). These bands are labeled in Fig. 1 as B, G, and R. As is seen from Fig. 1, the G and R bands are not observed in the spectrum of pure PVA, thus, it is natural to assume that they stem from the nanocomposites. The B band can be ascribed to the traces of the PVA emission that originates from the matrix of the nanocomposite. It is seen that with the change of NPs concentration the intensities of the bands as well as their spectral positions change. To analyze the spectral components in more detail, the deconvolution of the PL spectra was done.

**Fig. 2:**
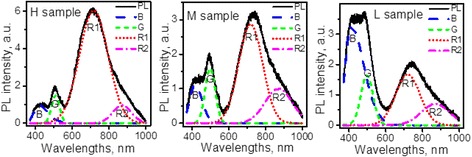
Deconvolution of the PL spectra: using four components with fixed spectral positions and linewidths

Figure 2 shows results of the deconvolution of the PL spectra of the samples H, M, and L. It is found that PL spectra of all samples in the whole spectral range can be fitted using four components: B, G, and two PL bands in the red and near infrared ranges that are labeled as R1 and R2. In view that the B band was presumed to be a residual emission from the polymeric matrix, its lineshape in the composite was chosen to be the same as in pure PVA (Fig. 1). All other components were fitted with a Gaussian lineshape. The choice of two components for the modeling of the R band was dictated by the necessity to fit its shift and extended low-energy wing.

The fitting revealed that with decreasing NPs concentration, the intensity of B band increases while the total intensity of other bands (G + R1 + R2) is reduced. The intensity of the R1 band strongly and monotonously decreases with the dilution while G band weakly increases. The R1 band intensity changes non-monotonously. The spectral positions of the G, R1, and R2 bands remain almost unchanged.

Thus, the fitting results show that the PL spectra can be described by four PL components which demonstrate different trends with decreasing NPs concentration.

### ODMR of Nanocomposites

**Fig. 3:**
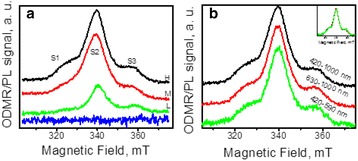
ODMR spectra. **a** ODMR spectra of the *L*, *M*, *H* composites and pure PVA detected in the whole spectral range **b** and the ODMR response of the *H* composite in different spectral ranges

To obtain additional information on the recombination processes in the nanocomposites we have done ODMR measurements. Figure 3a shows ODMR spectra of the H, M, and L samples as well as of pure PVA registered in the spectral range of 420–1000 nm. Note that pure PVA does not exhibit any ODMR signal while all CdS/PVA samples yield positive three-peak ODMR signal (the lines are labeled in Fig. 3a below as S1, S2, and S3). The intensity of the total ODMR signal diminishes with decreasing NPs concentration in the composites. Notably, the amplitude of the ODMR signal achieves ~ 1.5% for the H composite that is a rather high value as compared to the typical ODMR signals in different semiconductor materials [15].

Another feature of the ODMR signal is its independence on the spectral range of registration. Figure 3b shows ODMR spectra of the sample H measured in different spectral intervals. These intervals were chosen to distinguish between contributions to the ODMR signal from the G band (420–590 nm) and the R1 and R2 bands (630–1000 nm). The ODMR spectrum measured in the whole spectral range (420–1000 nm) is shown in Fig. 3b, for a comparison. The inset in Fig. 3b confirms the invariability of the ODMR signals in different spectral ranges: being normalized all three spectra coincide within the experimental accuracy. From the comparison of the ODMR spectra from H, M and L samples (Fig. 3a) it is also clearly seen that the shape of ODMR spectrum depends on the concentration of CdS NPs in the composites. The relative intensity of the S1 signal decreases with the dilution of the initial colloidal solution. It is accompanied by the narrowing of the S2 signal, and its shift towards higher magnetic fields. The S3 signal is the same in the H and M samples, while in the L sample it is observed at a higher magnetic field. However, the overlap between the signals complicates the detailed comparison of the ODMR spectra between different composites. To reveal the concentration-related changes in the ODMR the deconvolution of the spectra was performed.

A satisfactory modeling of the spectra can be obtained if four Gaussians are used. One Gaussian was introduced to fit the S1 signal; its relative intensity decreased with the decrease of the NPs concentration. Two Gaussians (the low-field and high-field components) were used to describe S2 signal and its shift toward higher magnetic field, in particular, this shift was modeled by diminishing of the low-field component contribution. One more Gaussian was applied to simulate S3 signal in the H and M composites while another one with a differed position was used to fit S3 signal in the L composite.

Analysis of this modeling revealed that the changes of Gaussian used to fit S1 signal and the low-field component of the S2 signal with varying NP concentration correlate. This finding allowed us to combine these two components into one asymmetric line (LK1) being characteristic for paramagnetic centers of axial symmetry in disordered systems. Thus, the number of the fitting components was reduced.

**Fig. 4:**
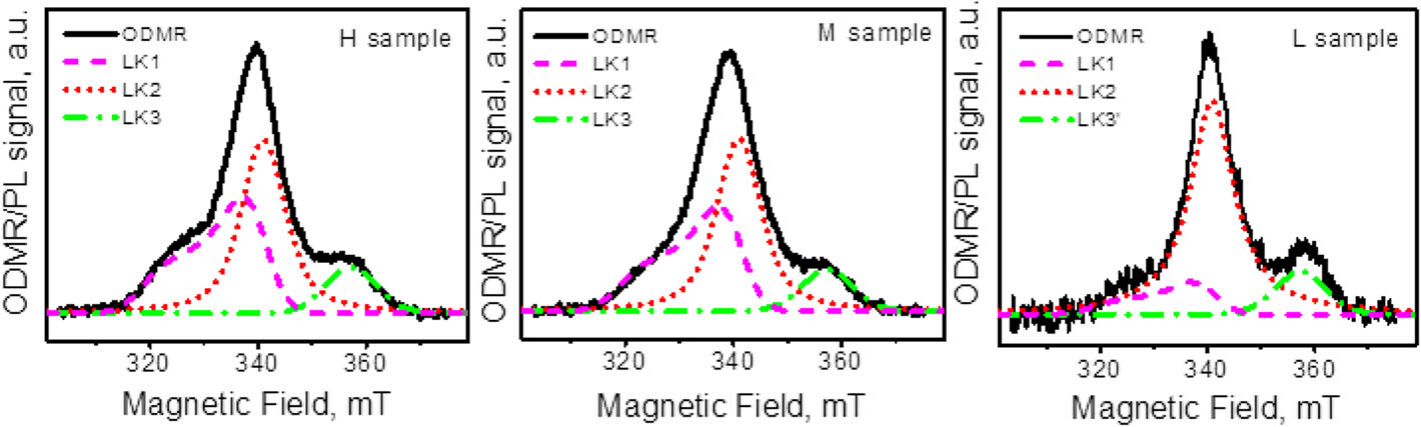
Fitting of the ODMR spectra. Fitting of the ODMR spectra of the composites by changing only the relative intensities of three components

The best fit of the experimental ODMR spectra was obtained using three following components (Fig. 4): for the composites H and M two symmetrical lines with *g* ~ 1.93, ΔH = 6 мT (LK2) and *g* ~ 1.85, ΔH = 5 мT (LK3) and one asymmetrical line with *g*||~ 2.075, *g*_⊥_ ~ 1.945, ΔH = 4 мT (LK1) while for the composite L the parameters of the second symmetric Gaussian are *g* ~ 1.83, ΔH = 5 мT (LK3’).

The LK1 and LK3 lines demonstrate different behavior with dilution. Namely, the relative intensity of LK1 monotonously decreases with decreasing NPs concentration while its position remains unchanged. On the other hand, the relative intensities of LK3 and LK3’ are almost the same while LK3’ is shifted to higher fields. So, as is seen from Fig. 4 the intensities of all fitting components vary independently for different samples, therefore, they should be attributed to different paramagnetic centers.

## Discussion

The PL emission of CdS/PVA nanocomposites has been extensively studied before [16–24]. Usually, very broad PL bands similar to the ones found in the present study are observed in blue, green, orange, and red ranges depending on the preparation conditions, chemicals used for NPs growth, size of NPs, etc. Despite of a large number of papers devoted to studying light-emitting properties of the CdS/polymer, the origin of PL bands is still under debate. For example, the high energy bands were ascribed either to excitonic and band edge emission or to donor-acceptor recombination. On the other hand, the low-energy bands were interpreted as being due to radiative recombination involving shallow and deep traps. Some of the PL bands were tentatively ascribed to surface (or interface) related recombination [23–27].

In the further analysis, we will consider nanocomposite as a combination of three constituents—the polymeric matrix, NPs, and interface. Accordingly, we will assume that the components of PL and ODMR signals can be related to either of these constituents. In this assumption, the anticipated behavior of individual components must be as follows:

Keeping in mind that the series of the samples under examination was formed by the dilution of the ripened colloid with the solution of pure PVA, and the body of NPs was not subjected to any changes, only the intensities of PL and ODMR components originating from the body of NPs can change concurrently with the content of NPs in the samples. The spectral positions and g-factors of the corresponding signals should remain unaltered.

The signals originating from the bulk of the matrix must also preserve their spectral positions and g-factors because the structure of the polymer would hardly feel decreasing the NPs concentration from 4 · 10^16^ to 1 · 10^16^ cm^−3^. However, even at these low concentrations, NPs can strongly influence the intensity of PL, as will be shown below.

Quite differently, the structure of the interfacial region can be changed by dissolution because the ratio between the number of NPs and number of polymeric macromolecules in the sample changes. Thus, the surrounding of NPs by polymer is altered, and the surface density of interface-related traps can be changed or even new types of interfacial centers can appear. These considerations lead us to the conclusion that the interface can be changed both qualitatively and quantitatively, which can alter both the positions and intensities of the PL and ODMR features.

In our samples, the fitting of the PL spectra demonstrates the presence of at least four bands, which exhibit different behavior with the decrease of the NPs concentration. The intensity of the B band, which was tentatively ascribed to the emission of polymer, increases with the dilution. This increase does not correlate with almost invariable matrix content in the composite (the share of PVA exceeds 99% in all samples). Nevertheless, this behavior is quite reasonable if one takes into account two processes that can diminish the matrix emission in nanocomposites. One of them is the reduction of polymer excitation due to competitive absorption of the incident light by the embedded CdS NPs, and another is the re-absorption of the B band emission by NPs. The larger concentration of NPs, the stronger both effects are. Thus, based on the described intensity behavior and spectral position we can state that the B band is related to the polymeric matrix. Therefore, the remaining (G + R1 + R2) signal definitely corresponds to the emissions from the NPs and/or interface, and its overall decrease with decreasing NPs concentration is quite consistent with this conclusion.

The G, R1, and R2 PL bands emerge at the same spectral positions in all samples. The invariability of spectral positions implies that the excited and ground states that are involved in these emissions are the same independently of the concentration. However, the efficiency of the corresponding emissions depends on the density of NPs in the samples as is seen from Fig. 2. Notably, the intensities of G, R1, and R2 bands do not change concurrently. The R1 band decreases approximately proportionally to the overall volume of NPs in composites. This behavior implies that all R1-related processes (excitation, relaxation, and recombination) can occur within the unchanged body of NPs. On the other hand, the total surface of NPs in the composites changes in the same manner as the overall volume (because the sizes of NPs do not change). Thus, it is not possible to attribute the R1-related processes unambiguously to the body or the surface of NPs. On the contrary, the G- and R2-related processes cannot be localized exclusively inside the NPs because the changes of their intensities do not correlate with the decrease of the NPs overall volume. Therefore, at least some of the processes that are involved in the G- and R2-emissions should occur at the interface. Thus, two of the four PL bands observed are interface-related and are changing together with interface modifications.

An additional information on the interface-related features of nanocomposites is obtained from ODMR results. Fortunately, the polymeric matrix does not give any ODMR signal. Therefore, the three lines that decrease with decreasing NPs concentration can be related only to paramagnetic centers located either in NPs volume or at the interface.

It should be noted that similar ODMR signals with three peaks have been observed before in the samples of CdS NPs incorporated in glass [28]. The shape of the spectra has been interpreted as a superposition of two signals: a singlet in the center and doublet on the wings of the spectrum. The origin of the singlet was explained by the presence of electron-hole pair with strong exchange interaction. This pair was assumed to be localized within the volume of CdS NPs. The doublet was also ascribed to the electron-hole pair located on the surface of CdS NPs with a weaker exchange interaction.

As seen from Fig. 4, the changes of LK1 and LK3 signals with the dilution of nanocomposites are not correlated, thus, in the case of CdS/PVA the signals should be treated as two independent signals. To the best of our knowledge, the centers with the similar spin-Hamiltonian parameters were not detected previously. Moreover, due to the absence of hyperfine structure, the identification of these centers is difficult. Nevertheless, some features revealed by fitting procedure (Fig. 4) provide a clue for the identification of the center related to the LK1 signal. As it was stated above, the correlation between the S1 Gaussian and the low-field component of the S2 signal allowed to combine them into a unified signal (LK1) from the anisotropic center with *g*||~2.075, *g*_⊥_ ~ 1.945, ΔH = 4 мT. In this spectral range, the surface O-related centers in CdS powders were registered by EPR method [29, 30]. Despite certain differences in the g-factors of the anisotropic signal detected and the centers reported in [29, 30], there are at least three reasons to assume that we also observe the O-related surface centers. The first reason is that different O-related centers have anisotropic g-factors with one component being close (or less) to the free electron *g* value (*g*_*e*_), while another one being much higher (for example *g*_||_ = 2.442 and *g*_⊥_ = 1.948 for the surface O_2_^-^ center in CdS; *g*_||_ = 1.972 and *g*_⊥_ = 2.309 for the surface O^-^ center in CdS powder [29]). The second reason is a very strong dependence of the *g* factors of the O-related centers on the surroundings (See, e.g., [31]). The third reason follows from the composition of the polymeric matrix. Chemical formula of PVA is [CH_2_CH(OH)]_n_, which means that there are plenty of oxygen atoms in the surroundings of the NPs. An additional proof of the polymer-related oxygen contribution to the LK1 signal is the absence of this signal in the ODMR spectra of nanocomposites consisting of CdS NPs in the matrix which does not contain oxygen other than unintended impurity (to be published later).

Two other single ODMR lines (LK2 and LK3) are not identified. However, it should be noted that their *g* factors are less than *g*_*e*_, therefore, most probably, these lines correspond to the donor-like paramagnetic centers. Notably, the *g* factor of LK3 changes with NPs concentration which points to the modification either of the structure of the corresponding paramagnetic center or its nearest surroundings. Thus, we tentatively ascribe this center to the interface.

The important feature of the ODMR spectra is the invariability of the signals at the registration in different (green and red) spectral regions (Fig. 3b). Such behavior is repeatedly observed for the centers of non-radiative recombination. However, the latter give negative ODMR signals, while the signals observed for our nanocomposites are positive. Positive sign of the ODMR that is observed for the studied CdS/PVA composites indicates unambiguously that we deal with paramagnetic centers participating in the sequence of processes that finally lead to the radiative recombination of the photo-excited carriers. However, spectral independence of the signals demonstrates that the paramagnetic centers registered are not the radiative ones. Thus, we conclude that all three ODMR signals correspond to the centers that form the energy levels involved into the processes of energy relaxation that occur before the radiative recombination. Notably, these levels are involved in both sequences of relaxation processes ending up with either green or red luminescence.

## Conclusions

The series of solid films of CdS/PVA nanocomposites with different interfacial conditions was fabricated. The state of interface was tuned by variation of the NPs surroundings via simple dilution of the as-synthesized colloid by its native polymer solution thus avoiding involving new chemical species. The dried composite films with different concentrations of NPs and, correspondingly, different surface density of passivating species surrounding a nanoparticle, demonstrated concentration-dependent spectroscopic properties that were analyzed by the PL and ODMR methods.

The PL spectra of all composites exhibited four broad emission bands. The high-energy band that is also observed in pure polymer was ascribed to the residual emission of the PVA matrix. The analysis of the bands emerging after the nanocomposite formation showed that at least two bands are interface-related.

Composite samples demonstrate a very strong ODMR signal while the reference PVA sample does not give any signal. The ODMR spectra showed three independent signals indicating the presence of one anisotropic (LK1) and two isotropic paramagnetic centers (LK2 and LK3). The sign of all ODMR features is positive. Two centers—LK1 and LK3—were shown to be interface-related.

The comparison between the PL and ODMR results show that all ODMR signals arise from the centers that form the energy levels which are involved into the processes of excited carriers relaxation before their radiative recombination. These processes precede the emission in green (G band) and red (both R1 and R2 bands) ranges.

## Abbreviations

CCD: Charged-coupled device
CdS: Cadmium sulfide
EPR: Electronic paramagnetic resonance
NP: Nanoparticle
ODMR: Optically detected magnetic resonance
PL: Photoluminescence
PVA: Polyvinyl alcohol

## References

[1] Jeon I-Y, Baek J-B (2010). Nanocomposites derived from polymers and inorganic nanoparticles. Materials.

[2] Kango S, Kalia S, Celli A, Njuguna J, Habibi Y, Kumar R (2013). Surface modification of inorganic nanoparticles for development of organic–inorganic nanocomposites - A review. Prog Polym Sci.

[3] Reiss P, Couderc E, De Girolamo J, Pron A (2011). Conjugated polymers/semiconductor nanocrystals hybrid materials -preparation, electrical transport properties and applications. Nanoscale.

[4] Bera D, Qian L, Tseng T-K, Holloway P (2010). Materials.

[5] Lodahl P (2012). A Volume in Woodhead Publishing Series in Electronic and Optical Materials.

[6] Zhou H, Liu J, Zhang S (2015). Quantum dot-based photoelectric conversion for biosensing applications. Trends in Anal Chem.

[7] Chin PTK, Hikmet RAM, Janssen RAJ (2008). Energy transfer in hybrid quantum dot light-emitting diodes. J Appl Phys.

[8] Cheng J, Wang S, Li X-Y, Yan Y, Yang S, Yang CL, Wang JN, Ge WK (2001). Fast interfacial charge separation in chemically hybridized CdS-PVK nanocomposites studied by photoluminescence and photoconductivity measurements. Chem Phys Lett.

[9] Rudko GY, Kovalchuk AO, Fediv VI, Ren Q, Chen WM, Buyanova IA, Pozina G (2013). Role of the host polymer matrix in light emission processes in nano-CdS/poly vinyl alcohol composite. Thin Solid Films.

[10] Greenham NC, Peng X, Alivisatos AP (1996). Charge separation and transport in conjugated-polymer/semiconductor-nanocrystal composites studied by photoluminescence quenching and photoconductivity. Phys Rev B.

[11] Sperling RA, Parak WJ (2010). Surface modification, functionalization and bioconjugation of colloidal inorganic nanoparticles. Phil Trans R Soc A.

[12] Kovalchuk AO, Rudko GY, Fediv VI, Gule EG (2015). Analysis of conditions for synthesis of CdS:Mn nanoparticles. Semiconductor Physics, Quantum Electronics & Optoelectronics.

[13] Mieloszyk J, Drabent R, Siodmiak J (1987). Phosphorescence and fluorescence of Poly(vinyl Alcohol) films. J Appl Polym Sci.

[14] Chen W, Huang G, Lu H, McCready DE, Joly AG, Bovin J-O (2006). Utilizing nanofabrication to construct strong, luminescent materials. Nanotechnology.

[15] Tretyak OV, Lvov VA, Barabanov OV (2002). Physical principles of spin electronics.

[16] Eychmuller A, Hasselbarth A, Katsikas L, Weller H (1991). Photochemistry of semiconductor colloids. 36. Fluorescence investigations on the nature of electron and hole traps in Q-sized colloidal CdS particles. BerBunsenGes Phys Chem.

[17] Ferrer JC, Salinas-Castillo A, Alonso JL, Fernandez de Avila S, Mallavia R (2009). Influence of SPP co-stabilizer on the optical properties of CdS quantum dots grown in PVA. Phys Procedia.

[18] Khanna PK, Gokhale RR, Subbarao VVVS, Singh N, Jun K-W, Das BK (2005). Synthesis and optical properties of CdS/PVA nanocomposites. Mater Chem Phys.

[19] Caraman I, Robu S, Gaşin P, Lazar I, Lazar G, Stamate M (2009). Photoluminescence properties of CdS/PVA nanocomposite thin films. Proc SPIE.

[20] Tomihira K, Kim DG, Nakayama M (2007). Photoluminescence dynamics of energy transfer between CdS quantum dots prepared by a colloidal method. JOL.

[21] Woggon U, Bogdanov SV, Wind O, Schlaad K-H, Pier H, Klingshirn C, Chatziagorastou P, Fritz HP (1993). Electro-optic properties of CdS embedded in a polymer. Phy Rev B.

[22] Wang H, Chen Z, Fang P, Wang S (2007). Synthesis, characterization and optical properties of hybridized CdS-PVA nanocomposites. Mater Chem Phys.

[23] Pattabi M, Saraswathi AB (2010). Optical Properties of CdS–PVA Nanocomposites. Composite Interfaces.

[24] Yoo DS, Ha SY, Kim IG, Choo MS, Kim KW, Lee ES (2011). Characterization of CdS nanoparticles embedded in polyvinyl alcohol. New Physics: Sae Mulli (The Korean Physical Society).

[25] Wang C-F, Xie H-Y, Cheng Y-P, Chen L, Hu MZ, Chen S (2011). Chemical synthesis and optical properties of CdS–poly(lactic acid) nanocomposites and their transparent fluorescent films. Colloid Polym Sci.

[26] Yamaki T, Asai K, Ishigure K, Sano K, Ema K (1999). DFWM study of thin films containing surface-modified CdS nanoparticles. Synth Met.

[27] Kumar S, Mehta SK (2015). Varying photoluminescence emission of CdS nanoparticles in aqueous medium: a comparative study on effect of surfactant structure. Nano-Structures & Nano-Objects.

[28] Lifshitz E, Litvin ID, Porteanu H, Lipovskii AA (1998). Magneto-optical properties of CdS nanoparticles embedded in phosphate glass. Chem Phys Lett.

[29] Sootna GD, Padam GK, Gupta SK (1979). ESR study of oxygen radicals formed in cadmium sulphide. Phys Status Solidi A.

[30] Miller DY, Haneman D (1971). Electron-paramagnetic-resonance study of clean and oxygen-exposed surfaces of GaAs, AlSb, and other III-V compounds. Phys Rev B.

[31] Vorona IP, Nosenko VV, Baran NP, Ishchenko SS, Lemishko SV, Zatovsky IV, Strutynska NY (2016). EPR study of radiation-induced defects in carbonate-containing hydroxyapatite annealed at high temperature. Radiat Meas.

